# Global pairwise RNA interaction landscapes reveal core features of protein recognition

**DOI:** 10.1038/s41467-018-04729-0

**Published:** 2018-06-28

**Authors:** Qin Zhou, Nikesh Kunder, José Alberto De la Paz, Alexandra E. Lasley, Vandita D. Bhat, Faruck Morcos, Zachary T. Campbell

**Affiliations:** 1 Department of Biological Sciences, University of Texas at Dallas, Richardson, TX 75080 USA; 2 Center for Systems Biology, University of Texas at Dallas, Richardson, TX 75080 USA

## Abstract

RNA–protein interactions permeate biology. Transcription, translation, and splicing all hinge on the recognition of structured RNA elements by RNA-binding proteins. Models of RNA–protein interactions are generally limited to short linear motifs and structures because of the vast sequence sampling required to access longer elements. Here, we develop an integrated approach that calculates global pairwise interaction scores from in vitro selection and high-throughput sequencing. We examine four RNA-binding proteins of phage, viral, and human origin. Our approach reveals regulatory motifs, discriminates between regulated and non-regulated RNAs within their native genomic context, and correctly predicts the consequence of mutational events on binding activity. We design binding elements that improve binding activity in cells and infer mutational pathways that reveal permissive versus disruptive evolutionary trajectories between regulated motifs. These coupling landscapes are broadly applicable for the discovery and characterization of protein–RNA recognition at single nucleotide resolution.

## Abstract (web summary)

RNA–protein interactions often depend on the recognition of extended RNA elements but the identification of these motifs is challenging. Here, the authors present a global integrated approach to analyze RNA–protein binding landscapes, mapping extended RNA interaction motifs for four RNA-binding proteins.

## Introduction

Proteins are integral to virtually every aspect of RNA function including: transcription, processing, localization, translation, and ultimately decay^1^. RNA-binding proteins (RBPs) associate with specific structures and sequences found in their regulatory targets. Their affinity for these elements confers regulatory potential and is a fundamental aspect of RNA control^2,3^. Accordingly, numerous next generation sequencing methods have emerged with the goal of understanding how proteins recognize their targets both biochemically and in cells^4–6^. These methods are complementary and have been reviewed elsewhere^4,6–11^. For instance, models that define key determinants of specificity from in vitro selection and high-throughput sequencing of RNAs (SEQRS) predict which bound sites facilitate regulatory encounters in vivo^12^. In this work, we integrate SEQRS with global probabilistic models to improve our understanding of how proteins associate with extended RNA elements.

Structured RNAs are integral components of ribosomes, spliceosomes, and telomerases^13^. Genome-wide surveys, which probe structure in vivo, enable unbiased detection of structured elements and suggest that structure profoundly impacts mRNA translation and stability^14–21^. Comparable advances in high-throughput biochemical approaches remain tenuous. For instance, genomic microfluidic analysis of a yeast RNA-binding protein captured a short known binding element but sampled only a reduced fraction of sequence space due to the compact nature of the host genome^22^. Microfluidic approaches have been proposed having the advantage of direct observation of binding events and accurate estimation of biochemical constants (see Ozer et al. as well as Greenleaf and co-workers)^7–9^. Sequencing methods can provide kinetic information and have been used to calculate enrichment scores that correlate with equilibrium dissociation constants^4,6,10^. A major challenge remains for the analysis of structured motifs. Methodological advances are needed to understand how proteins recognize these complex yet vital regulatory elements.

High-throughput methods, including SEQRS, induce selection to extract biochemical information. As a result of positive selection imposed by RNA interacting with RBPs, high-affinity RNA elements survive and thrive while those sequences that fail to interact are depleted^23,24^. One way to analyze the selection process is through the use of high-throughput sequencing and local models^25–27^. These local models are robust for identification of short motifs (typically 6–10 bases), but limited in analysis of extended binding elements. Because it is impractical to achieve a sufficient sequencing depth to capture all possible permutations of a short random sequence (~16 bases with existing capabilities), enrichment is scored for subsets of the total library size (typically 6–10 bases). Although the frequency of motifs in SEQRS provides a proxy for potential binding elements, precise native binding sites may not be the most frequently observed. Global models of binding may enable detection of biologically important sites. To enable analysis of extended binding elements, we developed a statistical framework for the generation of global statistical models based on direct coupling analysis (DCA)^28,29^. DCA has revealed key mechanistic insights into protein evolution, structure, dynamics, and function^28–37^. Our hypothesis is that pairwise interactions between individual RNA bases within a random library would reveal the underpinnings of motif recognition by RBPs.

Our approach yields a comprehensive view of specificity that we refer to as a DCA-scape. This method differs from positional weight matrix (PWM) models, which form the basis for most motif discovery algorithms^38^. PWMs are limited by the assumption of independence between different nucleotide positions of the binding site^39^. The DCA-scape captures both the identity of single sites and examines pairwise nucleotide interactions termed couplings across the complete site. Related approaches have yielded key insights into recognition of transcription factor-binding elements^40,41^. To achieve this inference procedure, we use DCA to estimate parameters in the nucleotide distribution originating from SEQRS data. Our approach expands on existing work on co-evolutionary signatures in RNA structure through experimental consideration of the selective force of protein binding on pairwise nucleotide couplings^42–44^.

Here, we infer DCA-scapes for P22N, λN, BIV TAT, and human TUT7. These are particularly challenging targets for unbiased analysis given their extended motifs, up to 28 bases, and preference for secondary structures. The DCA-scapes accurately determine biologically functional regions within their endogenous genomic contexts, predicts alternative sequences that preserve or enhance recognition, quantify evolutionary trajectories, and recapitulate past findings and suggest recognition motifs that might play important biological roles in bacterial and viral genomes.

## Results

### An integrated approach to analyze RNA interaction landscapes

**Fig. 1:**
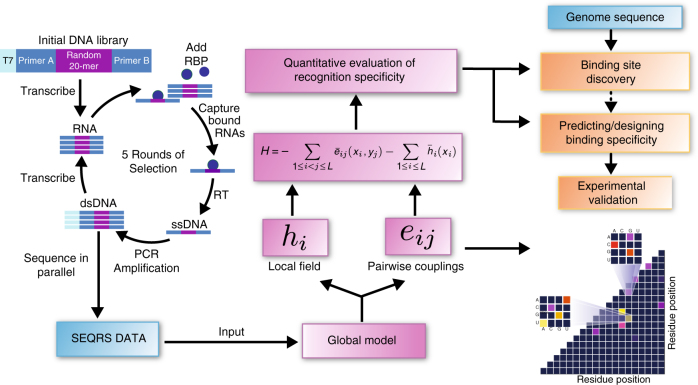
DCA-scapes provide global models of RNA recognition. RNA-binding proteins are incubated with libraries of in vitro transcribed RNA containing a 20 base random region (scheme adapted from ref. ^46^). RNA–protein complexes are isolated through washing and reverse transcribed to cDNA. Transcription adapters are attached to the library using PCR and the selection process is repeated for five rounds. After selection, the pool is subjected to high-throughput sequencing. The 20-mer region is used as an input for the creation of a global model. The model considers both the local nucleotide propensities and pairwise couplings to calculate a Hamiltonian score. These scores can be used to detect binding sites and predict the outcome of specific mutations. Pairwise interactions are visualized on a landscape and provide hints of molecular interactions relevant for recognition

There are two main elements to our method. First, we perform in vitro selection and high-throughput sequencing, and second, generate a global statistical model of the resulting data (Fig. 1). This allows us to sparsely sample the functional sequence space experimentally and then use those signals to computationally estimate the much larger space of functional sequences. Briefly, the SEQRS procedure begins with in vitro transcription of DNA oligonucleotides encoding a random 20-mer region^45,46^. The resulting pool of RNAs is incubated with purified recombinant protein immobilized on magnetic resin. After repeated washing, bound RNAs are thermally eluted and converted into double-stranded DNA using reverse transcriptase and PCR. This enrichment procedure is repeated for five cycles. Sequencing adapters and unique barcodes are added prior to high-throughput sequencing. The use of barcodes enables sequencing of multiple samples in parallel, and enables deconvolution of multiplexed data.

**Fig. 2:**
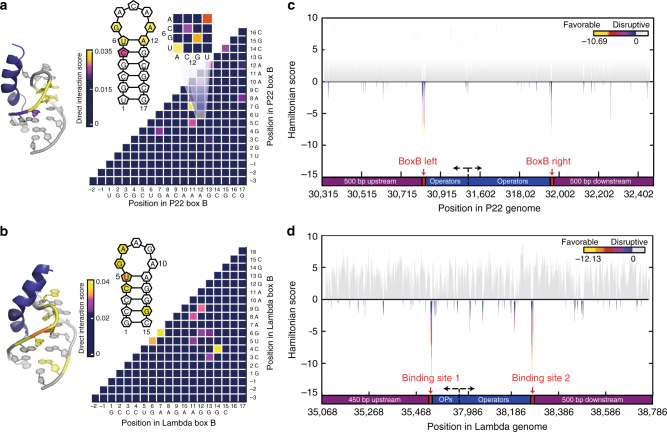
DCA-scapes of N proteins reveal known sites of association in vivo. **a** The structure of P22 N (PDB 1A4T [https://www.rcsb.org/structure/1A4T]) bound to box B motif is shaded according to direct information (DI) score. Yellow bases have the highest score while darker colors denote lower scores. A cartoon of the box B structure is shown to indicate the position of shaded sites in the 3D coordinates. The DI landscape indicates coupled relationships in the box B motif. **b** The structure of λ N (PDB 1QFQ [https://www.rcsb.org/structure/1QFQ]) bound to box B, a cartoon indicating specific coupling interactions observed on the DI landscape. **c** Discovery of in vivo-binding sites for P22 N based on Hamiltonian scores. Hamiltonian scores (*Y*-axis) across the genomic region encompassing the operator region are calculated based on estimated global probability parameters using DCA and provide a quantitative evaluation of binding in different genomic regions. Negative values indicate a higher probability of association and are shaded according to the inset color bar. **d** Predicted binding sites for λ N following the same procedure as in **c**

**Fig. 3:**
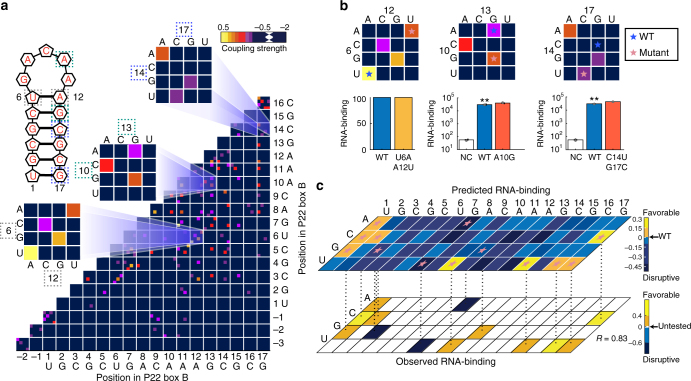
The P22N DCA-scape elucidates recognition of the Box B left element. **a** The P22 N protein-binding recognition landscape. Interactions are shaded according to the inset color scale. Representative interactions are enlarged to indicate predicted mutational outcomes. **b** Experimental confirmation of alternative specificities. The wild-type Box B left sequence is indicated with a blue star while mutants are labeled with pink stars. The U6A A12U mutation was previously described as an alternate binding mode and is predicted to result in a favorable interaction (6C–12C is observed in the wild-type Box B right motif). The A10G single mutant and C14U, G17C double mutant were analyzed using the yeast-three hybrid assay (error bars represent 1 standard deviation, three biological replicates corresponding to separate cultures were conducted for each measurement, **represents *p*-value < 0.01 under one-tailed *t*-test). Both mutations predicted to be favorable retain binding. **c** A mutational landscape of the Box B left sequence. The upper panel illustrates the predicted binding specificity matrix for all possible single mutations. Red stars indicate substitutions that were analyzed experimentally. The lower panel provides the experimentally observed binding activities collected based on these predictions (see Supplementary Table 1). Boxes are colored by the relative change using the wild type sequence as a point of reference. The landscape accurately captures both disruptive and favorable changes in protein–RNA recognition between protein P22 and the structured RNAs (six biological replicates corresponding to separate cultures were conducted for each measurement)

The second element involves generation of the statistical model. The DCA-scape is a model that contains three types of metrics: direct information (DI) pairs, coupling landscapes, and Hamiltonian scores. In the first stage of analysis, sequencing data are used to estimate, for positions *i*, *j* and nucleotide *x*, pairwise couplings *e*_*ij*_(*x*_*i*_, *x*_*j*_) and local biases *h*_*i*_(*x*_*i*_) that are parameters of a joint probability distribution of 20-mers, i.e. *P*(*x*_1_,…, *x*_*L*_) with *L* = 20 (Fig. 1). These parameters are used to compute DI for all possible pairwise interactions between individual bases. DI values among pairs provide a broad quantitative measure of pairwise interactions. DI values calculated for other systems (e.g. amino acid sequences in protein families) serve as a proxy to detect coevolving residues that are in direct physical contact^31^. Here, we examine if these pairs are informative in the context of RNA–protein interactions (Fig. 2a, b). DCA-scapes also provide coupling values that are represented on a landscape to visualize interacting pairs of nucleotides. Unlike DI values, pairwise coupling landscapes take into account nucleotide composition and provide a finer scale picture of the strength of nucleotide connectivity, see the expanded cells in Fig. 3a. The third element of the DCA-scape, the Hamiltonian score, is a global sum of recognition parameters (pairwise couplings) *e*_*ij*_(*x*_*i*_, *x*_*j*_) and also local biases *h*_*i*_(*x*_*i*_) for all possible interactions in a given sequence that exists in the complete sequence space of a random RNA library. This score quantitatively predicts how likely a given protein associates with a particular RNA sequence and provides a sequence-dependent global model of specificity. For this model to be useful, it would ideally predict endogenous biologically functional interactions across entire genomes, faithfully recapitulate biophysical interactions, and provide a rational for mutational pathways. We evaluate these applications in the context of DCA-scapes for four distinct RBPs.

### Pairwise couplings elucidate regulatory elements

As a test of the accuracy of DCA-scapes, we examine the anti-termination N proteins encoded by phage λ and P22. The symmetric binding sites, termed nut-boxB RNAs, flank the operator sequence and serve as a key function in transcription^47^. After SEQRS, we compared our experimentally derived Hamiltonian scores to a random distribution (Supplementary Fig. 1). The positive end of the experimentally derived Hamiltonian scores distribution forms a normal-like distribution (0–10 range), which is similar to a random distribution, which suggests some non-specific binders that are independent from each other and do not affect the robustness of couplings estimation. The difference between the two distributions suggests that the experiment has identified sequences that are distinct and may preferentially associate with λ and P22 N. To compare these scores to sites of productive binding in vivo, Hamiltonian scores were calculated for genomic regions flanking the operator regions of P22 and λ N arranged in the order in which they are transcribed (Fig. 2c, d). In both cases, the dominant valley (negative values indicate an increased probability of interaction) is situated directly on known binding elements (P22 nut-boxB right *p*-value = 7.3×10^−12^, P22 nut-boxB left *p*-value = 3.2 × 10^−13^, λ nut-boxB right *p*-value = 3.5 × 10^−13^, and λ nut-boxB left *p*-value = 6.3 × 10^−15^ under one-tailed *z*-test). We expanded our analysis to encompass the entire genome of phage λ and P22, and found the same conclusion (Supplementary Fig. 2). The highest-ranking sequence of λ genome, as judged by the Hamiltonian score, is situated at the precise site of the known binding site (AUC = 0.937, FDR~10^110^). P22 N binding elements are found in the top 10 Hamiltonian scores sequences in its genome (AUC = 0.9923, FDR~10^−12^) (Supplementary Data 2). There are seven out of 41,705 sequences that have a better Hamiltonian score than the binding elements. Five of the seven sequences lack secondary structure. We tested the binding affinity of these seven sequences and confirmed that those are false positives (Supplementary Fig. 7). We conclude that the DCA-scape is sufficiently sensitive to detect endogenous binding elements in vivo.

To better understand the linkage between bases after selection with λ N and P22N, we generated landscapes using DI (Fig. 2a, b). To rationalize these relationships suggested by the landscape, we examined biophysical models derived from NMR measurements^48,49^. A priori, at least two non-mutually exclusive types of interaction contribute toward a strong coupling score. These are nucleotide interactions that preserve secondary structures and RNA-protein interactions that impart specificity between related secondary structures. We see instances of both. For example, positions 6 and 12 (P22 Box B), or 5 and 11 (λ Box B) appear to maintain stem structures. Conversely, residues situated in loop regions (P22 RNA residue 7 and 11, λ RNA residues 6 and 7) appear to impart specificity through loop specific contacts. Residue 4 and 7 of P22 RNA are on the same side of the RNA proximal to P22 N protein, a coupling that may arise as the result of contributions to protein recognition. In the case of λ RNA base 6 is vital as it is strongly coupled to base 5, 7, 12, and 13. Base 6 resides in a loop region while its interacting partners are located in structured and unstructured portions of the RNA. Consistent with the notion that base 6 is key for recognition; substitution of G6 with A6 abolishes λ N binding^50^. The information obtained through DI identifies RNA bases involved in maintaining RNA secondary structure and protein interactions.

### DCA-scapes elucidate high-resolution maps of specificity

To understand the contribution of couplings between individual RNA bases with additional precision, we analyze pairwise coupling values for all possible base pairs at each position in the P22 N motif (Fig. 3a). Each unique potential interaction is shown on a pairwise landscape matrix along each axis. As a point of reference, the wild-type boxB left composition and structure is shown as a cartoon and listed along each axis. This element is well represented on the landscape, interestingly; there are additional coupling relationships that depart from the sequence specified by the consensus element. In the context of the BoxB sequences, interactions that maintain the stem structure in the wild-type element (3C–15G, 5C–13G, and 6U–12A for left, 6C–12C for right) have strong coupling scores consistent with the consensus motif. However, we also observe alternative pairings. For example, mutations which alter the identity of bases 6 and 12 yet preserve base pairing are well tolerated^51^ (Fig. 3b, left). This suggests that the stem structure is important for recognition, yet, these positions permit other nucleotide combinations^51^. Remarkably, several bases that do not appear to interact directly with one another (10A–13G and 3C–6U) but are found in the wild-type element are strongly coupled. Mutation of base 10 from A to G shows higher coupling in the landscape than the wild type. We tested this experimentally and observed that this change slightly enhances binding suggestive of a key role in facilitating protein recognition (*p*-value = 0.0079, under one-tailed *t*-test) (Fig. 3b, middle). Indeed, the loop portion of the RNA has been shown to be crucial for N protein recognition^51^. The landscape suggests that two stem bases (14C–17G) are not optimal for binding. We tested this notion with a substitution to a combination with a higher coupling score—14U 17C (Fig. 3b, right). We observed a small enhancement in binding relative to the wild-type element (*p*-value = 0.0024, under one-tailed *t*-test). These observations suggest that the DCA-scape is an informative source of alternate nucleotide sequences that maintain protein binding.

As a comprehensive test for the utility of DCA-scapes for tailored RNA recognition, we predicted three classes of mutations in the Box B left element with respect to their effects on protein binding: enhancers, inhibitors, or those with no effect (Fig. 3c). Having predicted the position of the Box B element in the genome using Hamiltonian scores, we reapplied this knowledge to rank all possible sequence permutations relative to the wild-type element (Fig. 3c). In yeast three-hybrid experiments, we probed nine positive, one wild-type-like, and three disruptive single mutations. All predictions in both classes were confirmed experimentally. The Hamiltonian score is uniformly able to predict mutations that stabilize protein binding for Box B left (Pearson’s *R* = 0.83) and Box B right (Pearson’s *R* = 0.67) (Supplementary Fig. 3, Supplementary Data 1). As an additional test of our model, we compared our predictions to activity assays that included more than one point mutation^51^ (Supplementary Table 1) and found a significant Pearson correlation of 0.69 (Supplementary Fig. 4a). We extended this analysis to also examine binding of λ N to the same mutant series. The Pearson correlation was also significant (0.78 Supplementary Fig. 4b). The major source of error in these comparisons was related to the magnitude of the effects as opposed to their directionality. Collectively, these experiments suggest that Hamiltonian scores have a strong predictive value for understanding the consequences of sequence variants.

As a more extreme test for the predictive power of the Hamiltonian scoring function, we mutated and tested RNAs with trajectories with single up to six mutations and their interaction with P22 N. These mutations were introduced throughout the RNA in stem and loop regions. We tested two trajectories where all of the substitutions were predicted for sequences that have favorable Hamiltonian scores as a proxy to retain binding and 10/12 predictions were accurate (Supplementary Fig. 5). Two cases (both quadruple mutants) where mutations disrupted two base-pairing interactions in the stem hindered protein binding. In both cases, RNAs with subsequent five or six mutations still interacted with P22 N. This suggests that the Hamiltonian score is able to accurately predict end points sequences for complex mutational trajectories that retain their protein binding ability.

### Evolutionary pathways between functional RNA elements

**Fig. 4:**
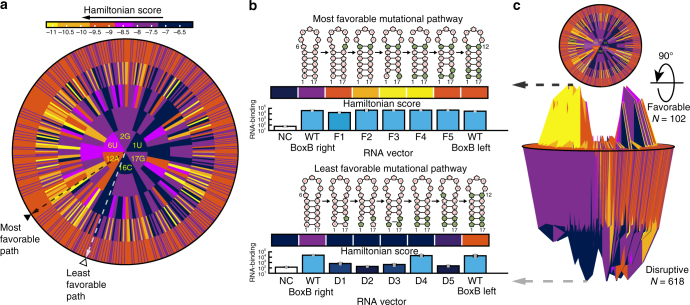
Mutational trajectories between two natural binding elements. **a** Predicted outcomes from mutations that bridge the Box B left and Box B right motifs. A Hamiltonian score estimates the predicted outcome from each mutation and each possible change at six positions is illustrated on a disc with five concentric circles radiating from Box B right. Light colors suggest favorable mutations while dark colors represent disruptive mutations that might block the evolutionary pathway according to the color scale bar. Two extreme pathways are labeled by dashed arrows (black for most favorable mutational pathway, white for least favorable mutational pathway). **b** Evaluation of mutational pathways in cells using the yeast-three hybrid assay (see Supplementary Data 1). Mutational pathways are represented with secondary RNA structures (upper for most favorable pathway, lower for least favorable pathway). Bars depict experimental outcomes. **c** Rotation of **a** provides a three-dimensional representation potential mutational trajectories. Valleys identify less favorable pathway while peaks suggest favorable ones (error bars represent 1 standard deviation, three or more biological replicates corresponding to separate cultures were conducted for each measurement)

Over the course of P22 phage evolution, two high-affinity binding elements with similar structures yet different sequence emerged as biological targets. Six positions in the stem regions differ between the structures. We sought to determine the entire landscape of mutations linking the two elements (Fig. 4a). Hamiltonian scores for box B right (origin) and box B left (outer ring) suggest that most pathways do not support interaction (darker regions in the landscape). Rotation of the plot along the abscissa reveals the complex landscape of RNA recognition (Fig. 4c). In the lateral view of Hamiltonian scores, trajectories form both valleys and mountains. Peaks represent favorable mutations that tend to be preferred in nature, while valleys are disruptive mutations that impair binding. We examined both the most and least favorable trajectories based on a Hamiltonian search. All of the optimal sequences preserved RNA-binding (black dashed arrow in Fig. 4a and upper panel in Fig. 4b). Conversely, 4/5 of the mutations on the unfavorable pathway eliminated recognition (gray dashed arrow in Fig. 4a and lower panel in Fig. 4b). We conclude that the mutational landscape surrounding two functional binding elements is complex and that the Hamiltonian-based landscape can suggest optimal mutational pathways with high accuracy.

### Extended binding elements in BIV-TAT

**Fig. 5:**
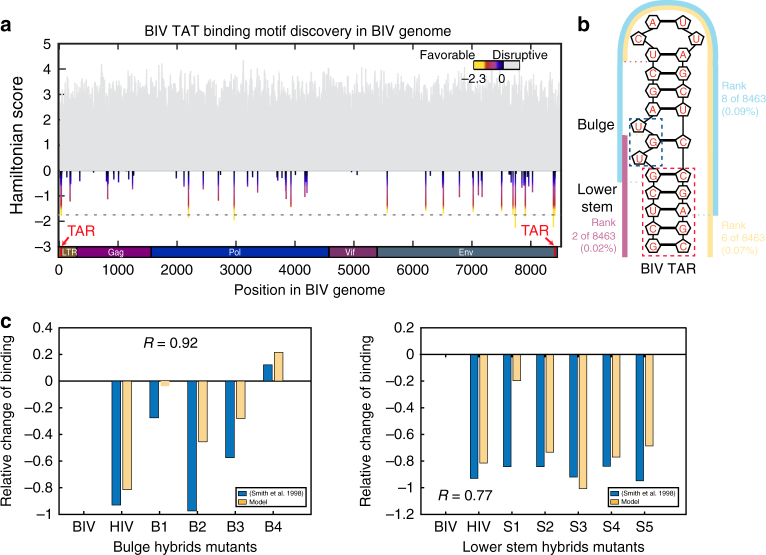
The BIV DCA-scape accurately predicts mutational outcomes. **a** Identification of trans-activating regions in the BIV genome (red arrows and dotted line indicate the positions of the binding elements). **b** The secondary structure of the BIV TAR RNA. The lower portion of the element containing an essential bulge has the most favorable Hamiltonian score. **c** A comparison of mutational assays in the TAR bugle (left, Pearson’s *R* = 0.93) and lower stem (right, Pearson’s *R* = 0.77) to Hamiltonian scores^55^

Bovine immunodeficiency virus (BIV) trans-activator of transcription (TAT) binds the trans-activation response (TAR) element. This interaction facilitates production of viral RNA and contributes to the replication cycle through recruitment of the P-TEFb elongation factor^52^. The 26-nucleotide TAR element is longer than the selection libraries utilized in SEQRS by six nucleotides. Thus, we analyzed this protein to determine if DCA-scapes were capable of extracting useful information from a library that is shorter than the native binding element. We reasoned that our approach would capture useful information as crucial interactions in BIV TAT–TAR binding are located predominantly on one end of the RNA—the bulge and lower stem regions (Fig. 5b)^53,54^. To determine the accuracy of our approach, we examined the entirety of the BIV genome (Fig. 5a). The two identical TAR elements present in the BIV genome were detected with a high degree of confidence (*p*-value = 2.7 × 10^−10,^ under one-tailed *z*-test). From the sequences with the top 10 Hamiltonian scores, five of them contain part of the binding element. The native full-length-binding element can be extracted by joining these sequences together (Fig. 5b). The Pearson correlation between Hamiltonian scores for BIV TAT and mutations that impact the bulge or stem regions was high, 0.93 and 0.77, respectively (Fig. 5c, supplementary Data 1)^55^. These data suggest that DCA-scapes are capable of re-capitulating known mutational and structural properties of extended RNA structures based on libraries with a fixed length shorter than known in vivo-binding elements.

### Recognition of pre-miRNAs by processing enzymes

Terminal uridylyl transferase 7 (TUT7) is a master regulator of microRNA (miRNA) biogenesis and function^56^. TUT7 binds to pre-miRNAs from the let-7 family and controls their biogenesis through uridylation^57^. The TUT7 RNA-binding region is similar in composition to highly basic viral RNA-binding domains^58^. We reasoned that the specificity of this domain would provide insight into how TUT-7 discriminates between pre-miRNAs and unrelated structures. We computed Hamiltonian scores for two mutant forms of a TUT7 target (Supplementary Fig. 6a). One version of pre-let-7a lacks the entire loop region, the other lacks part of the stem region. Specificity scores for binding of RNAs derived from the unmodified (*p*-value = 0.0046, under one-tailed *z*-test) and the mutant with a stem deletion (*p*-value = 0.00078, under one-tailed *z*-test) suggest potential for binding. However, deletion of the loop eliminated this possibility (*p*-value = 0.098, under one-tailed *z*-test). Consistent with this series of predictions, only the unmodified and stem deletion RNAs were uridylated by TUT7^57^. Given that little is known about TUT7 specificity, we confirmed binding of the TUT7 RNA-binding region to a top scoring sequence using florescence polarization (Supplementary Fig. 6b). Our coupling landscapes are able to accurately predict binding of RNAs of a related sequence that possess major differences in structure and length. Our findings suggest a molecular basis for the difference in enzymatic activity observed with TUT7 on model substrates from the let-7 family.

## Discussion

Our study explores the specificity of RBPs for complex RNA targets for four different systems. Structured RNAs are particularly important given their use as tethers in heterologous assays, potential therapeutic utility, aptamer applications, and biological prevalence^17,18,59–64^. We analyzed prokaryotic and eukaryotic viral RBPs and a human enzyme as these domains bind structured RNAs. Our approach provides a means to simultaneously probe protein–RNA recognition in a space of 4^20^ sequence combinations in a single experiment. This expands on related microfluidic methods, which access ≈10^7^ permutations^9^. A pairwise global mathematical model of the sampling in this enormous space deals effectively with the sparsity of experimental data and estimates parameters to dissect and engineer specificity for a variety of systems. This work demonstrates that sequence coupling models can be applied to understand protein–RNA recognition from a synergy of theory and high-throughput experiments. Our studies permit three major conclusions. First, we found that Hamiltonian scores were remarkably sensitive for identification of endogenous binding sites, which have apparently evolved to form high affinity interactions. Although we focused on the viral sites of recognition, this approach could be used to score binding elements in any genome. As P22N, and λ N are commonly used as tethers for synthetic biology applications in eukaryotic cells, identification of their off target binding elements in these contexts could be helpful in experimental design. Moreover, the BIV homolog HIV TAT binds host RNA species at 2074 genomic regions in immune cells^65^. Our approach provides a means to predict these interactions based on biochemical information with a high degree of accuracy.

Second, the data we have generated enable the use of RNA variants corresponding to a range of different binding activities. In particular, we have generated variants with improvements in binding which are particularly desirable. When we incorporate structural information into our analysis, we are able to separate effects on RNA structure from those that originate from contributions to protein recognition. Similar results have been obtained for the MS2 coat protein based on variants of the consensus binding element^8^. It appears, based on our analysis of multiple systems, that both mechanisms are broadly required for recognition of structured RNA elements.

Finally, we developed an approach to analyze possible evolutionary bridges between two functional binding elements for P22N and validated the pathways experimentally. We find that both pathways resulted in intermediates that had destabilized secondary structures. In the case of the most favorable route, deleterious substitutions can be compensated for based on their introduction in a favorable order. This suggests that while there are many ways in which two structures could be linked through a mutational process, the sequence in which these changes occur is vital for preservation of binding. Thus, DCA-scapes can be applied to propose possible evolutionary trajectories at single nucleotide resolution and to predict the effects of sequential mutations on binding activity. Our technology focuses on structured RNAs but the approach is general and should also be applicable to RBPs that bind linear elements.

## Methods

### SEQRS

Recombinant proteins were generated through amplification with the primer sets described in Supplementary Table 2 with GoTaq (Promega). All clones were validated with Sanger sequencing prior to expression in BL21 codon plus cells as GST fusions. The protein constructs used were P22N (NAKTRRHERRRKLAIER), λ N (MDAQTRRRERRAEKQAQWKAAN), BIV TAT (RPRGTRGKGRRIRR), and a 90 amino acid stretch of human TUT7 (residues 1362–1452). Prior to selection, the initial RNA library was generated using the primer sets described in Supplementary Table 2 using GoTaq (Promega). Following recovery with the GeneJET PCR Purification Kit, transcription of 1 μg of dsDNA was conducted using the AmpliScribe T7-Flash Transcription Kit (Epicentre)^45^. Removal of DNA was accomplished through incubation with Turbo DNAse. Recombinant proteins were immobilized on magnetic resin. As a competitor, 200 ng of RNA was added to each of the binding reactions (Ambion). Each reaction contained protein and 100 μl of SEQRS buffer—50 mM Tris–HCl pH 8, 0.1 mM ZnCl_2_, 150 mM NaCl, 0.1 mM MgCl_2_, 0.1% NP-40, 0.5% glycerol, 200 ng yeast tRNA competitor, and 0.1 units of RNase inhibitor (Promega). Samples were incubated for 30 min at 22 °C prior to magnetic isolation of protein–RNA complexes. Unbound RNAs were aspirated and the beads were subjected to four washes with 200 μl of SEQRS buffer. After the final wash step, resin was suspended in elution buffer (1 mM Tris pH 8.0) containing 10 pmol of the reverse transcription primer 1615. Samples were heated to 65 °C for 10 min and then cooled on ice. Reverse transcription was conducted with ImProm-II reverse transcription reaction (Promega). The ssDNA product was used as a template for 25 cycles of PCR using a 50 μl GoTaq reaction (Promega).

### Data processing

The 20 base pair sequencing reads (*L* = 20) from SEQRS were arranged in *M* rows (*M* = 1,579,936 in P22 N, *M* = 1,165,352 for λ N and *M* = 1,447,349 for BIV TAT). As a negative control, GST immobilized on resin was used to compute a background. The sequence array can be represented with the following notation:

1 $$A = \left( {A_i^a} \right),i = 1, \ldots ,L,a = 1, \ldots ,M$$

where four types of nucleotides were translated into consecutive numbers 1, 2, 3, 4. A version of DCA^31^ was developed to analyze RNA datasets for both protein-binding target sequences, as well as background negative control. The final landscape of protein recognition or DCA-scape was estimated by subtracting the background parameters from the target protein recognition preference parameters (see Eq. 4 in the following section).

### DCA and Hamiltonian scores

DCA is an unbiased, global inference methodology used to infer joint probability distributions from sequence data. DCA has been used widely to study evolutionarily related sequences with applications to structural and system biology^28–31^. Here, rather than studying families of proteins we analyze nucleotide sequences obtained experimentally after a stage of selection towards RBP binding (SEQRS). In this work, DCA infers a joint probability distribution of RNA fragments (20-mers). The parameters of this distribution include pairwise elements (couplings) and single site occupancies (local fields). The coupling parameters provide information about non-local interactions of nucleotide positions. Such interactions include Watson–Crick pairs but also non-trivial connections that contribute to protein-binding specificity and recognition.

In order to infer a global joint probability distribution to satisfy the statistical observations for input RNA sequences, marginal empirical frequency counts are generated to be consistent with input data. Derived from the maximum-entropy principle, this global probability distribution can be modeled with an explicit mathematical form similar to a Boltzmann distribution^31^. Two types of parameters are estimated for this distribution: pairwise couplings *e*_*ij*_(*x*_*i*_, *x*_*j*_) and local biases (fields) *h*_*i*_(*x*_*i*_). In DCA for protein families, typically, couplings and fields are set to 0 relative to gaps while SEQRS output sequences contain no gaps. Therefore, during the inference process, to reduce the freedom from independent constraints, we set all couplings and fields measured relative to one nucleotide (*N*_*k*_) to 0 for each round where *N*_*k*_∈{*A*, *C*, *G*, *T*/*U*}:

2 $$e_{ij}( {x_i,x_j = N_k} ) = e_{ij}( {x_i = N_k,x_j} ) = h_i\left( {x_i = N_k} \right) = 0$$

For each round, pairwise couplings *e*_*ij*_(*x*_*i*_, *x*_*j*_) and local fields *h*_*i*_(*x*_*i*_) are estimated with respect to each state (*A*, *C*, *G*, *T/U)*^31^. Finally, parameters $\overline {e_{ij}} ( {x_i,x_j} )$ and $\overline {h_i} \left( {x_i} \right)$ are obtained by calculating the mean of the couplings and local fields for each of the four nucleotide gauged states. These parameters inferred from the input binding sequences can be collectively interpreted as a fitness function or Hamiltonian:

3 $$H(x_1, \ldots ,x_L) = - \mathop {\sum }\limits_{1 \le {\mathrm{i}} < j \le L} \overline {e_{ij}} \left( {x_i,x_j} \right) - \mathop {\sum }\limits_{1 \le i \le L} \overline {h_i} \left( {x_i} \right)$$

Hamiltonians can be calculated for the binding preference data and the experimental control data (bg). The effective Hamiltonian is defined as

4 $$H_{{\mathrm{eff}}}\left( \sigma \right) = H\left( \sigma \right) - H_{{\mathrm{bg}}}\left( \sigma \right)$$

where *σ* is the vector representing the nucleotide sequence that is the target of a RBP. The more negative a Hamiltonian score is, the more favorable is the protein–RNA-binding specificity.

### Genome-wide binding site discovery

Binding specificities of genome sequences can be quantified with the Hamiltonian metric defined in the previous section. We obtained genome sequences from NCBI: λ genome (NC_001416.1); P22 genome (NC_002371.2); and the BIV genome (NC_001413.1). With a sliding window of size 20, starting from the first nucleotide to the end of the genome, binding specificities of genome sequences are quantified with Hamiltonian scores. By ranking effective Hamiltonian scores and their *p*-values for different sequence regions, binding motif sites can be identified with high accuracy (see Fig. 2, Supplementary Fig. 2).

### Null models and *p*-values

To compare and evaluate the binding strength of genome sequences or mutants at a binding motif, we created 100 million random 20-mers sequences to build a null model. These random sequences are generated in such a way to keep the same ACGU proportion as the specific genome of interest. Hamiltonian scores of these random sequences are calculated according to the parameters learned from each specific protein-binding preference data (Supplementary Fig. 1). Based on the Hamiltonian score distribution of such random sequences, *p*-values for Hamiltonian scores are estimated using the one tail *z*-test. False discovery rates (FDR) and *Q* values are estimated from the *p*-values by utilizing the procedure introduced by Storey^66^.

### DI pairs and DCA-scapes

Based on the parameters estimated for the joint probability distribution of RNA sequences, a quantification of how two sites in the RNA are directly coupled can be deduced. We use the DI formulation^31^, which computes the Kullback–Leibler divergence between the joint frequency counts and the joint pairwise distributions obtained from the parameters inferred by DCA. The pairwise couplings landscapes (DCA-scapes) directly illustrate the pairwise couplings information for each nucleotide combination between pairs of positions as an 80 × 80 matrix. This matrix is organized in 20 × 20 matrices with 4 × 4 submatrices including all nucleotide pairs for a given position pair *i*, *j* (see Fig. 3). The DCA-scapes and the DI pairs provide a quantitative description of protein–RNA recognition and help us characterize the functional variability of a given interface.

### Mutational effects and Hamiltonian search optimization

In order to evaluate or design alternative-binding specificities of protein recognition, we generate a list of sequences, which cover possible mutations: single mutants (Fig. 3) to multiple mutations (Supplementary Figs. 4, 5) with respect to the native recognition RNA motifs. We then quantify these mutants using Hamiltonian scores and search for sequences that convey a desired property (low values for favorable binding and high values for disruptive effects). The candidates then are validated experimentally to test the accuracy of the model. This procedure has low computational complexity and can be used to screen a large number of mutants in silico.

### Fluorescence polarization assays

Recombinant TUT7 (1362–1452) was incubated with Cy5 5′ labeled RNA of the sequence GCAGUCUUAACGCUGCCUUA. Binding was conducted in 30 μl of 45 mM Tris–HCl pH 8.0, 90 mM NaCl, 0.2% Tween-20 at 22 °C for 60 min. Measurements were collected in triplicate using a 96-well Tecan plate reader. Non-linear least-squares regression analysis was conducted using Kaleidagraph (Synergy Software)^67^.

### Yeast three-hybrid assays

Binding activity assays were conducted with a single copy of the P22N sequence (NAKTRRHERRRKLAIER) fused to the DNA-binding domain^68,69^. Subcloning was achieved using the primer sets described in Supplementary Table 2. Luminescence data were collected using the β-Glo reagent (Promega) and measured with a 96-well Tecan plate reader.

### Code availability

The code used to compute DCA-scapes is available to the scientific community online at: http://morcoslab.org/?page_id=385.

### Data availability

The sequence data that support the findings of this study are available from datadryad.org with the identifier doi:10.5061/dryad.qs734tp. Raw data corresponding to the figures can be found in Supplementary Data File 1. All other data supporting the findings of this study are available from the corresponding authors on reasonable request.

## Electronic supplementary material

Supplementary Information

Description of Additional Supplementary Files

Supplementary Dataset 1

Supplementary Dataset 2
