# Supplementary material for: Global pairwise RNA interaction landscapes reveal core features of protein recognition
Source: Nat Commun. 2018 Jun 28;9:2511. doi: 10.1038/s41467-018-04729-0 (PMC6023938; doi:10.1038/s41467-018-04729-0)
Supplement: Supplementary file 1 — Supplementary Information [file 41467_2018_4729_MOESM1_ESM.pdf]

Supplementary Figures

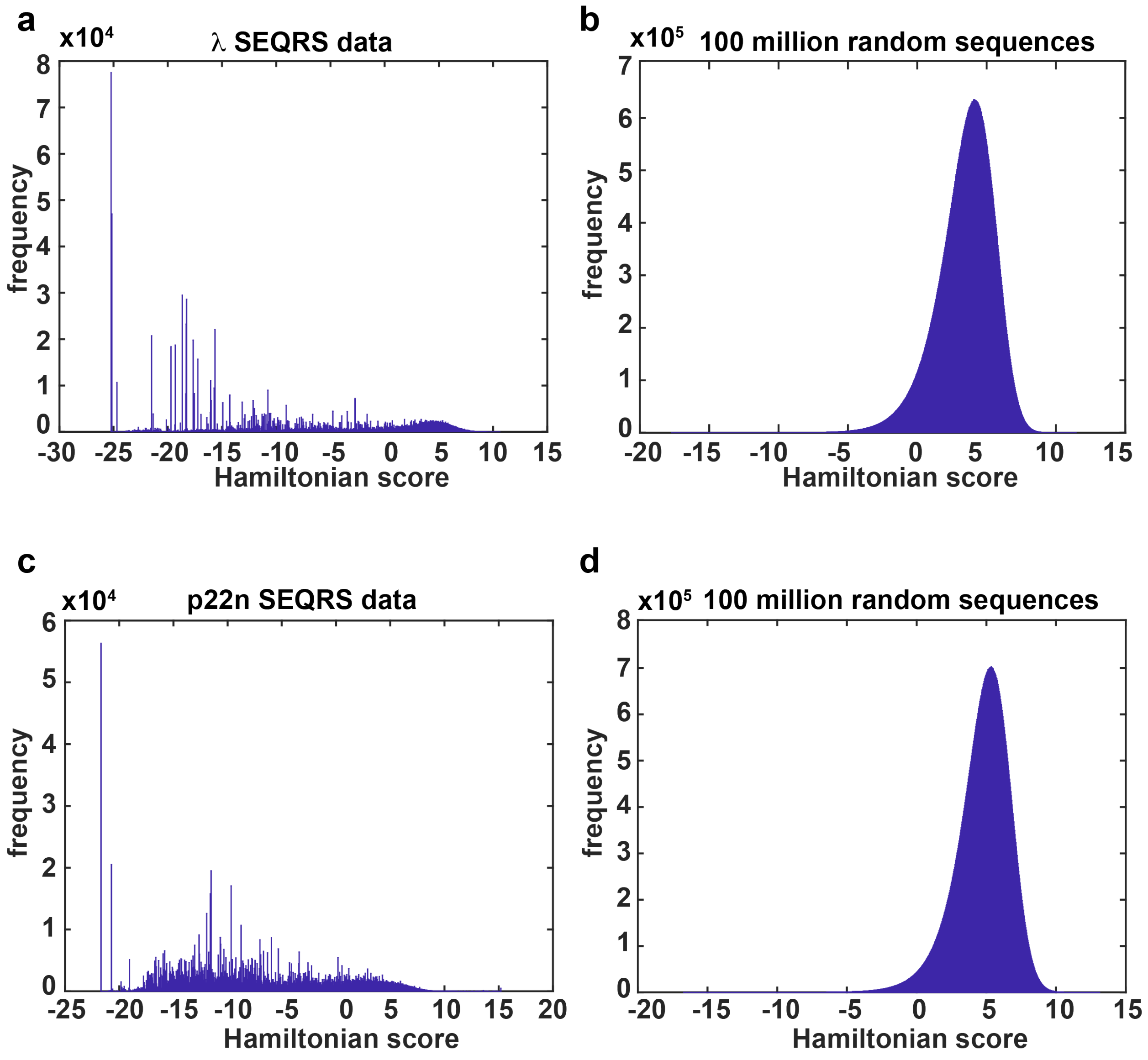

Supplementary Figure 1 - The distribution of Hamiltonian scores for sequencing data differs from simulated random data. The distribution of frequency (y-axis) of Hamiltonian scores (x-axis) in two datasets. (a) Hamiltonian scores based on SEQRS were calculated for λ N. (b) Distribution of 100 million random sequences with similar nucleotide composition to the λ genome. (c) Hamiltonian scores based on SEQRS data for P22 N. (d) Hamiltonian scores for 100 million random sequences with a similar nucleotide bias to the P22 genome.

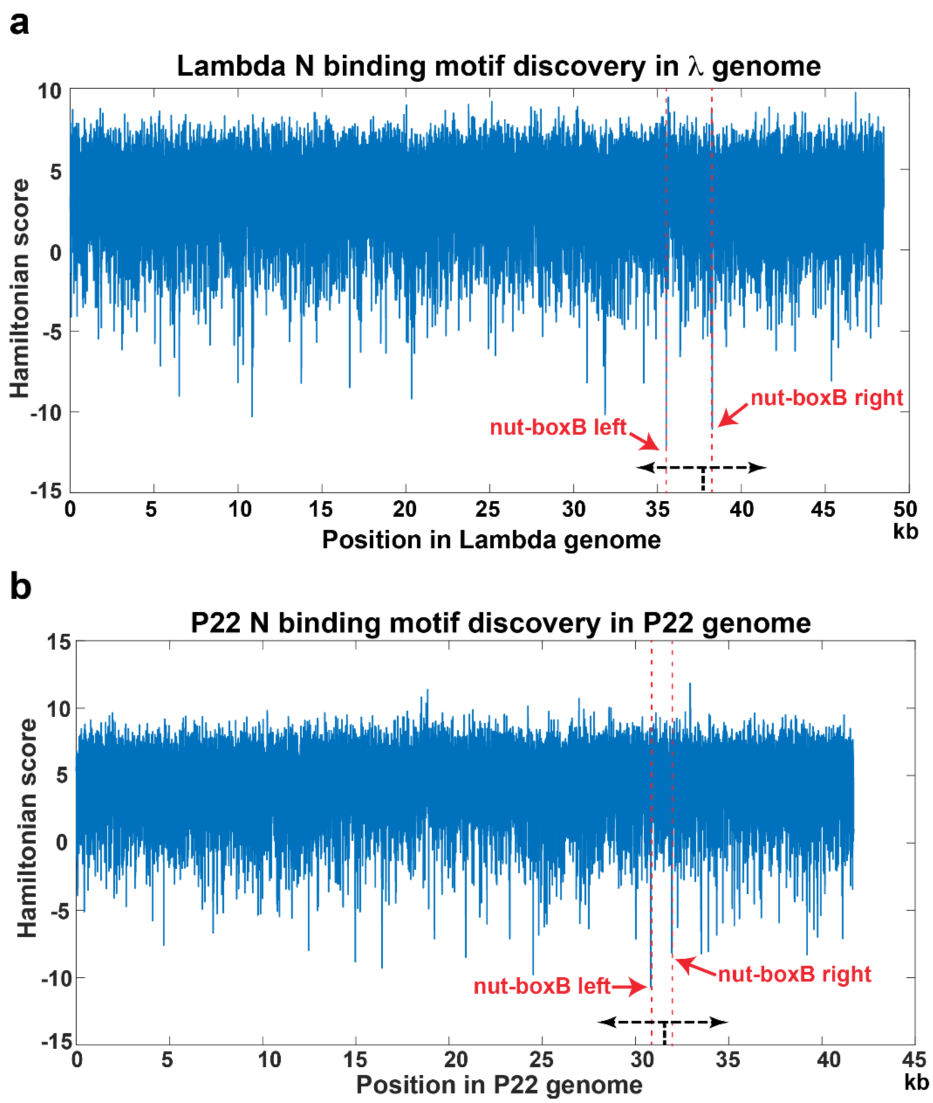

Supplementary Figure 2- DCA-scapes predict protein binding sites across the entire genome. (a) Predicted binding sites of λ N protein across the entire λ genome. The x-axis indicates one orientation of the λ genome. The y-axis corresponds to Hamiltonian scores. The positions of the Box B elements are indicated with red arrows. (b) P22 N binding sites identification across the entire p22 genome.

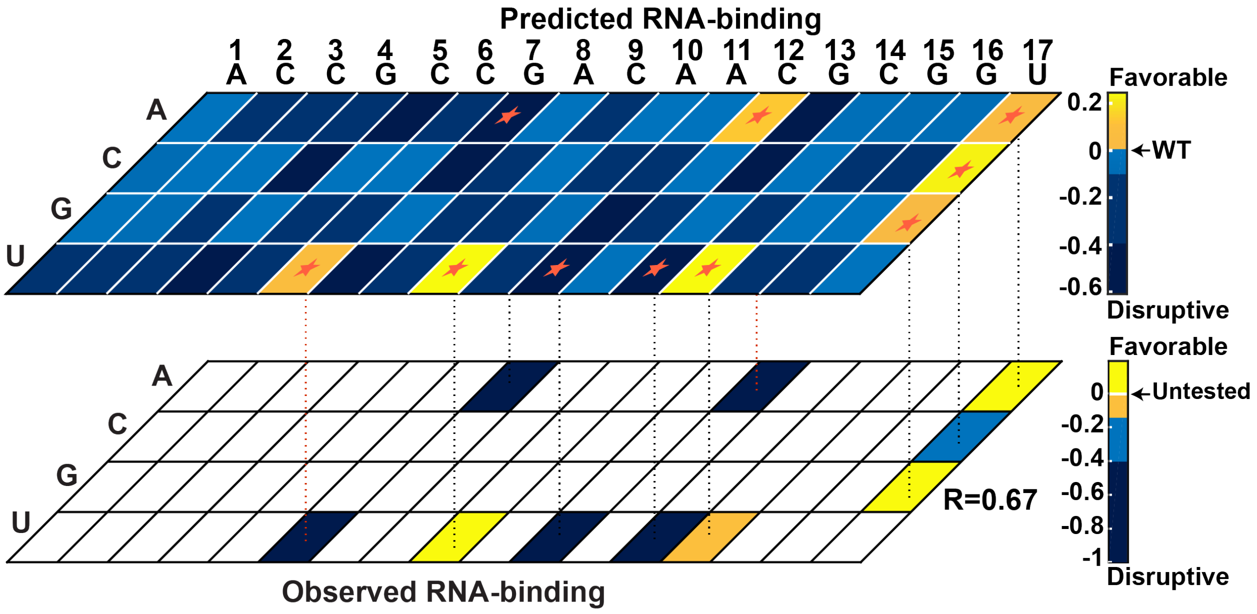

Supplementary Figure 3 - Model validation: predictions versus corresponding binding activities in the P22 BoxB right element. The upper panel illustrates the predicted binding specificity matrix for all possible single mutations. Red stars indicate substitutions that were analyzed experimentally. The lower panel provides the experimentally observed binding activities collected based on these predictions (number of experimental replicates = 3). Boxes are colored based on their relative binding with respect to the wild-type sequence.

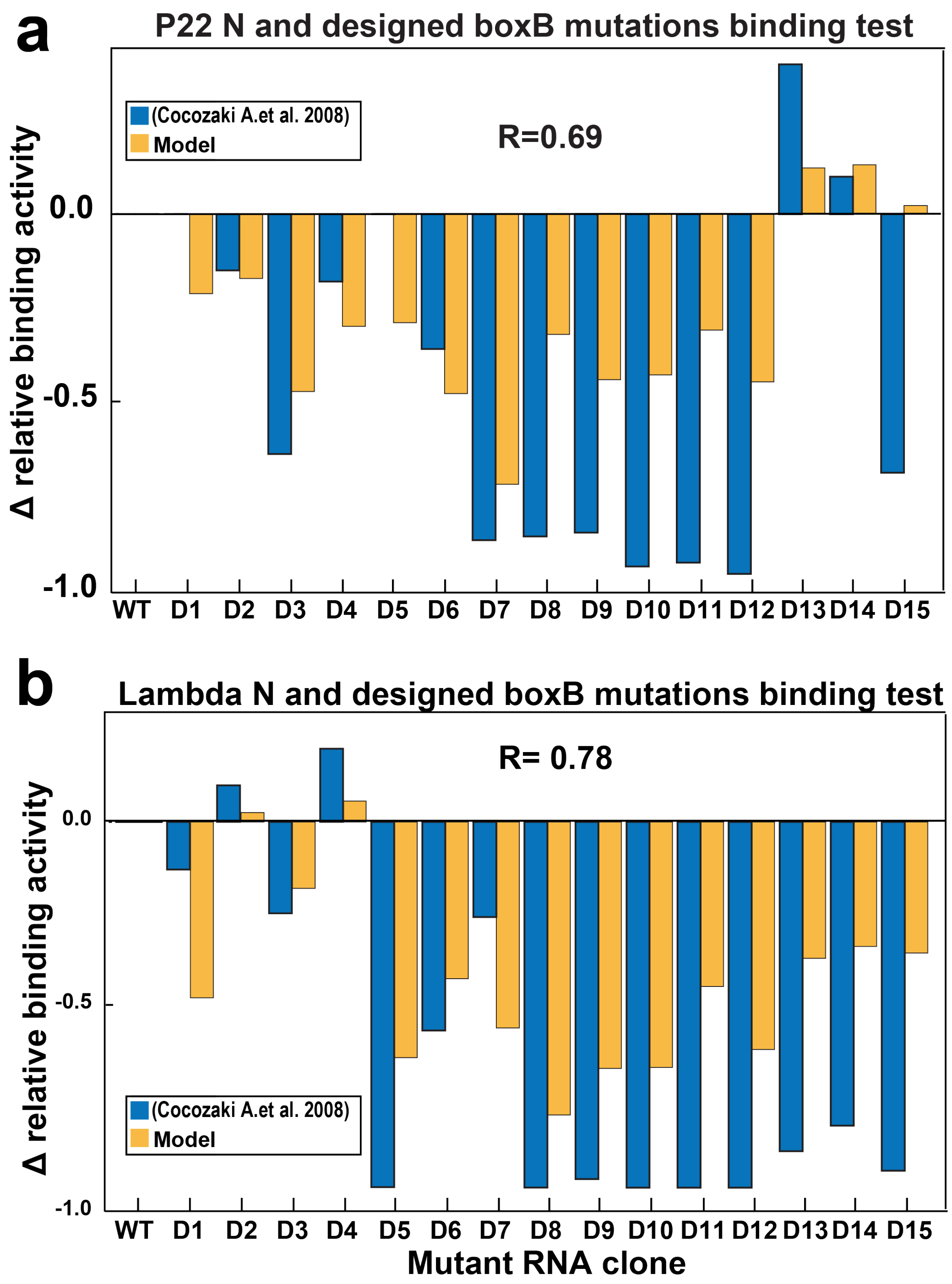

Supplementary Figure 4 - DCA-scapes correlate with binding activity measurements. Comparisons to prior mutational studies for P22 N protein (a) and λN protein. (b) Yellow bars indicate Hamiltonian values. Blue bars are the relevant RNA-binding assay responses[1]. Pearson correlation coefficients (R) are shown at the top of each panel.

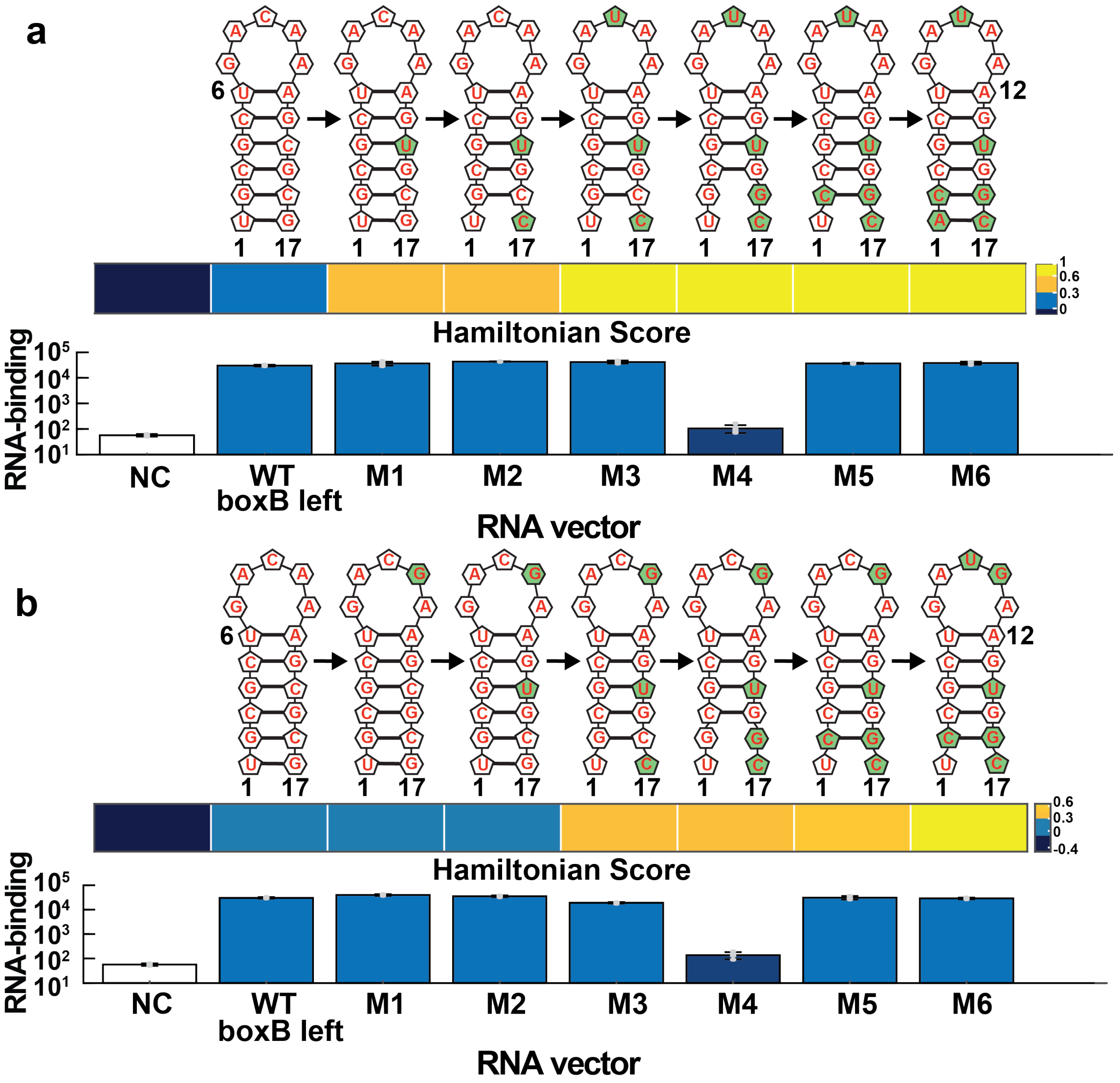

Supplementary Figure 5 - Design and optimization of progressive mutational pathways by DCA-scapes. Binding assays through six sequential mutations representing all possible nucleotides in the Box B left RNA sequence were compared with Hamiltonian scores. Two lists of mutations were tested and validated by three hybrid systems: (a) Mutants with increasing number of mutations (shown in secondary structures) retain functional binding except for a quadruple mutant (M4) which is then rescued by a fifth mutation. (b) A second example of functional mutant trajectories discovered using DCA-scapes. (error bars represent 1 s.d., experimental replicates = 3).

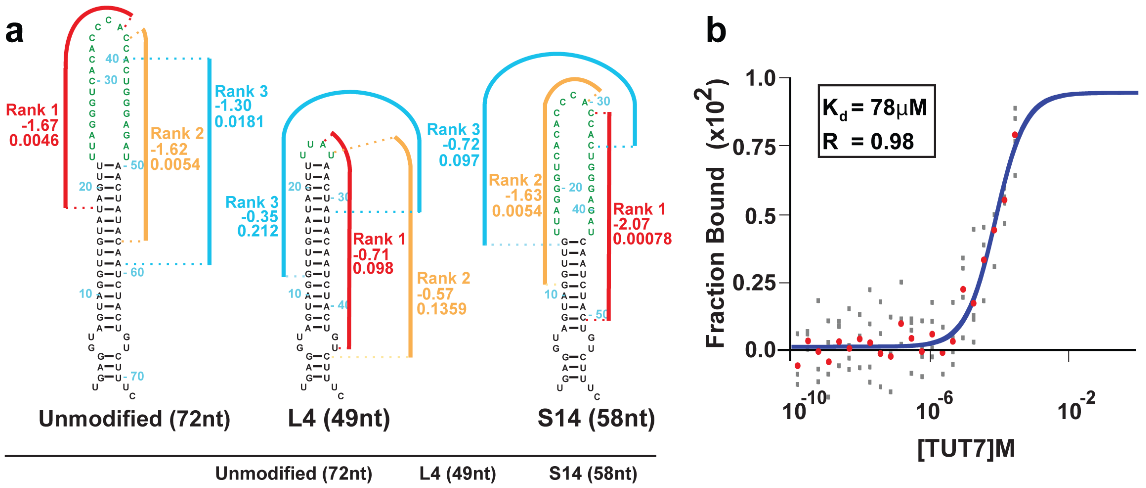

	Unmodified (72nt)	L4 (49nt)	S14 (58nt)
Hamiltonian score	-1.67	-0.71	-2.07
p-values	0.0046	0.098	0.00078
Uridylation (Kim B. et al. 2015)	yes	no	yes

Supplementary Figure 6 - Characterization of TUT7 recognition of RNA. (a) DCA-scapes predicted the binding specificities for unmodified pre-let-7*a*-1, the loop deletion mutant (L4) and a stem deletion mutant (S14). Red lines indicate the strongest Hamiltonian score sequence region for each RNA (with rank, Hamiltonian score and *p*-value). The predictions were validated by an *in vitro* uridylation assay[2]. (b) Equilibrium dissociation constants for TUT7 binding to the top scoring Hamiltonian sequence (GCAGUCUUAACGCUGCCUUA) were obtained using florescence polarization. Red dots show averages of three technical replicates (grey dots). Non-linear least squares regression analysis was used (Pearson's R = 0.98) [3].

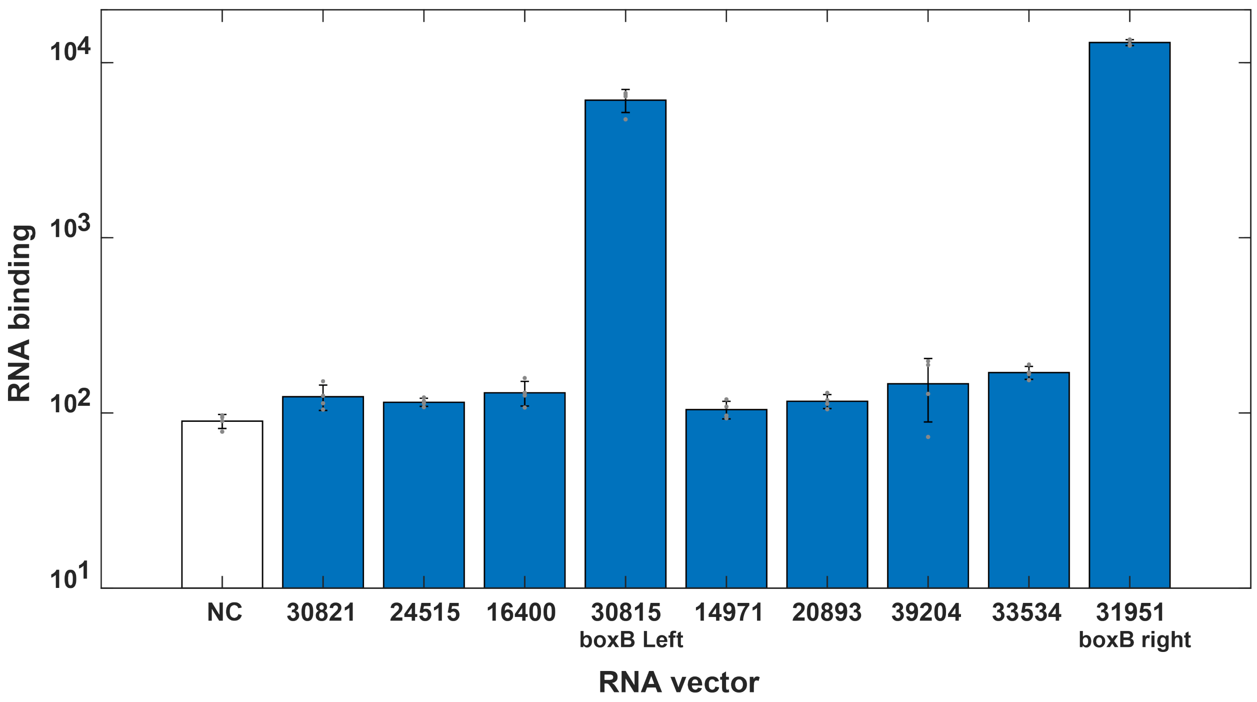

Supplementary Figure 7 - RNA binding affinity of top sequences in P22 genome. Seven out of 41,705 sequences have Hamiltonian scores comparable to known binding elements. Binding assays conducted in the yeast-three hybrid system suggest they are false positives. Most of these sequences are not predicted to form secondary structures (error bars represent 1 s.d., experimental replicates = 4).

Supplementary Tables

Binding activity of designed boxB mutants

Name	Sequences	% of Experiment Binding activities		Hamiltonian scores	
		P22 N	λ N	P22 N	λ N
P22 $boxB_{left}$	UGCGCUGACAAAGCGCG	100	—	-9.57	—
λ $boxB_{right}$	GCCCUGAAAAAAGGGCA	—	100	—	-11.28
D1	UGCGCUGGCAAAGCGCG	100	87	-7.55	-5.86
D2	UGCGCUGAAAAAGCGCG	85	110	-7.92	-11.57
D3	UGCGCUGGAAAAGCGCG	36	75	-5.04	-9.23
D4	UGCGCUGAAGAAGCGCG	82	120	-6.71	-11.92
D5	UGCGCAGACAAUGCGCG	100	0.2	-6.80	-4.01
D6	UGCGCAGAAAAUGCGCG	64	43	-4.99	-6.45
D7	UGCGCAGGAAAUGCGCG	13	74	-2.67	-4.93
D8	UGCGCCGACAACGCGCG	14	0.07	-6.50	-2.25
D9	UGCGCCGACAAGGCGCG	15	2.4	-5.34	-3.68
D10	UGCGCGGACAACGCGCG	5.7	0.06	-5.47	-3.71
D11	UGCGCUGACAAGGCGCG	7.3	0.06	-6.61	-6.20
D12	UGCGCGGACAAUGCGCG	4.3	0.05	-5.29	-4.27
D13	ACCGCUGACAAAGCGGU	140	10	-10.76	-7.08
D14	UCCGCUGACAAAGCGGG	110	17	10.84	-7.44
D15	AGCGCUGACAAAGCGCU	31	4.7	-9.80	-7.24

Supplementary Table 1 - Model predictions and binding activity assays for 15 alternative sequences in λ N and P22 N. A comparison of predicted binding patterns for λ N and P22 N to cell based binding activity assays[1]. Underlined nucleotides indicate base substitutions for the native P22 box B left RNA. For each protein (λ N or P22 N), the binding activity ratio of alternative sequences were calculated relative to the cognate RNA sequences (P22 box B left for P22N and λ box B right for λ N protein). Graphical versions of these data appear in Supplementary Figure 4.

Primer Name	Purpose	Primer Sequence
1619	Library generation	CTGATCCTACCATCCGTGCTNNNNNNNNNNNNNNNNNNNNCACAGCTTCGTACCGAGCGG
1614	Reverse transcription primer	CTGATCCTACCATCCGTGCT
1615	Amplification primer (contains T7)	TAATACGACTCACTATAGGGCCGCTCGGTACGAAGCTGTG
1616	Illumina adapter primer	AATGATACGGCGACCACCGAGATCTGCTCTTCCGATCTCTGATCCTACCATCCGTGCT
1618	Illumina adapter primer (X is the sample barcode)	CAAGCAGAAGACGGCATACGAGAT XXXXXX TCTTCCGATCCCGCTCGGTACGAAGCTGTG
1617	Custom sequencing primer	GCTCTTCCGATCTCTGATCCTACCATCCGTGCT
P22N peptide	Generation of P22N	CGCCCGCGTGGTACTCGTGGAAAGGGGCGTCGTATTCGCCGC
P22N F	Cloning of P22N into pGex6P-1 digested with XhoI	TGTTCCAGGGGCCCCTGGGATCCCCGGAATTCCCGGGTCGACTCCGCCCGCGTGGTACTC
P22N R	Cloning of P22N into pGex6P-1 digested with XhoI	CCGAAACGCGCGAGGCAGATCGTCAGTCAGTCACGATGCGGCCGCTCTAGCGGCGAATACGACGCC
lambda N peptide	Generation of lambda N	ATGGACGCTCAGACTCGTCGTCGTGAACGCCGCGCGGAGAAACAGGCTCAGTGGAAGGCGGCAAAT
lambda N F	Cloning of lambda N into pGEX6P-1 digested with XhoI	TGTTCCAGGGGCCCCTGGGATCCCCGGAATTCCCGGGTCGACTCATGGACGCACAGACCCGC
lambda N R	Cloning of lambda N into pGEX6P-1 digested with XhoI	CCGAAACGCGCGAGGCAGATCGTCAGTCAGTCACGATGCGGCCGCTCTAATTTGCCGCCTTCCACTGAG
BIV TAT peptide	Generation of BIV TAT	CGCCCTCGTGGCACCCGTGGGAAAGGTCGCCGTATCCGCCGC
BIV TAT F	Cloning BIV TAT into pGEX6P-1 digested with XhoI	TGTTCCAGGGGCCCCTGGGATCCCCGGAATTCCCGGGTCGACTCCGCCCTCGTGGCACCCG
BIV TAT R	Cloning BIV TAT into pGEX6P-1 digested with XhoI	CCGAAACGCGCGAGGCAGATCGTCAGTCAGTCACGATGCGGCCGCTCTAGCGGCGGATACGGCGA
TUT-7 F	Cloning TUT-7 into pGEX6P-1 digested with XhoI	TGTTCCAGGGGCCCCTGGGATCCCCGGAATTCCCGGGTCGACTCATGAGGAGAAAAGTAAGACGGCGG
TUT-7 R	Cloning TUT-7 into pGEX6P-1 digested with XhoI	CCGAAACGCGCGAGGCAGATCGTCAGTCAGTCACGATGCGGCCGCTCTAACGTTTTTCTCTTAAGTCTTTGTCAT
Swap pGEX-pGAD F	Subcloning of pGEX constructs into pGADT7 cut w/ECORI	TACGACGTACCAGATTACGCTCATATGGCCATGGAGGCCAGTGAA CCG GAA TTC CCG GGT CGA CTC
Swap pGEX-pGAD R	Subcloning of pGEX constructs into pGADT7 cut w/ ECORI	AGCTCGAGCTCGATGGATCCCGTATCGATGCCCACCCGGGTGGAAGTCACGATGCGGCCGCTCTA
pGEX 5'	pGEX sequencing	GGGCTGGCAAGCCACGTTTGGTG
T7	pGAD T7 sequencing	TAATACGACTCACTATAGGG

Supplementary Table 2 – Primer sets. These primers were used for SEQRS, cloning of protein expression vectors, generation of activation domain fusion proteins, and sequence validation respectively.

Supplementary References

1. Cocozaki, A.I., Ghattas, I.R. & Smith, C.A. Bacteriophage P22 antitermination boxB sequence requirements are complex and overlap with those of lambda. *J Bacteriol* **190**, 4263-4271 (2008).
2. Kim, B. et al. TUT7 controls the fate of precursor microRNAs by using three different uridylation mechanisms. *EMBO J* **34**, 1801-1815 (2015).
3. Campbell, Z.T. et al. Identification of a conserved interface between PUF and CPEB proteins. *J Biol Chem* **287**, 18854-18862 (2012).
