# Supplementary material for: Global pairwise RNA interaction landscapes reveal core features of protein recognition
Source: Nat Commun. 2018 Jun 28;9:2511. doi: 10.1038/s41467-018-04729-0 (PMC6023938; doi:10.1038/s41467-018-04729-0)
Supplement: Supplementary file 2 — Description of Additional Supplementary Files [file 41467_2018_4729_MOESM2_ESM.docx]

**Description of Additional Supplementary Files**

**File Name:** Supplementary data 1

**Description:** Experimental data. Tab 1 contains the P22 Box B left single mutations relevant to figure 3. Tab 2 contains the P22 Box B right single mutation relevant to figure 3. Tab 3 contains the mutational pathway data relevant to figure 4. Tab 4 contains the measurements of BIV mutants relevant to figure 5.

**File Name:** Supplementary data 2

**Description:** P-values, false discovery rates (FDR) and Q value for binding site predictions made in entire genomes using Hamiltonian scores for λ N, P22 N and BIV TAT.
